# Clustered Regularly Interspaced Short Palindromic Repeats Are *emm* Type-Specific in Highly Prevalent Group A Streptococci

**DOI:** 10.1371/journal.pone.0145223

**Published:** 2015-12-28

**Authors:** Po-Xing Zheng, Yuen-Chi Chan, Chien-Shun Chiou, Chuan Chiang-Ni, Shu-Ying Wang, Pei-Jane Tsai, Woei-Jer Chuang, Yee-Shin Lin, Ching-Chuan Liu, Jiunn-Jong Wu

**Affiliations:** 1 Institute of Basic Medical Sciences, College of Medicine, National Cheng Kung University, Tainan, Taiwan; 2 Department of Medical Laboratory Science and Biotechnology, College of Medicine, National Cheng Kung University, Tainan, Taiwan; 3 Center for Research, Diagnostics and Vaccine Development, Centers for Disease Control, Taichung, Taiwan; 4 Department of Microbiology and Immunology, College of Medicine, Chang Gung University, Tao-Yuan, Taiwan; 5 Department of Microbiology and Immunology, College of Medicine, National Cheng Kung University, Tainan, Taiwan; 6 Department of Biochemistry and Molecular Biology, College of Medicine, National Cheng Kung University, Tainan, Taiwan; 7 Department of Pediatrics, College of Medicine, National Cheng Kung University, Tainan, Taiwan; 8 Center of Infectious Disease and Signaling Research, National Cheng Kung University, Tainan, Taiwan; 9 Department of Biotechnology and Laboratory Science in Medicine, School of Biomedical Science and Engineering, National Yang-Ming University, Taipei, Taiwan; Faculdade de Medicina de Lisboa, PORTUGAL

## Abstract

Clustered regularly interspaced short palindromic repeats (CRISPR) are the bacterial adaptive immune system against foreign nucleic acids. Given the variable nature of CRISPR, it could be a good marker for molecular epidemiology. Group A streptococcus is one of the major human pathogens. It has two CRISPR loci, including CRISPR01 and CRISPR02. The aim of this study was to analyze the distribution of CRISPR-associated gene cassettes (*cas*) and CRISPR arrays in highly prevalent *emm* types. The *cas* cassette and CRISPR array in two CRISPR loci were analyzed in a total of 332 strains, including *emm*1, *emm*3, *emm*4, *emm*12, and *emm*28 strains. The CRISPR type was defined by the spacer content of each CRISPR array. All strains had at least one *cas* cassette or CRISPR array. More than 90% of the spacers were found in one *emm* type, specifically. Comparing the consistency between *emm* and CRISPR types by Simpson’s index of diversity and the adjusted Wallace coefficient, CRISPR01 type was concordant to *emm* type, and CRISPR02 showed unidirectional congruence to *emm* type, suggesting that at least for the majority of isolates causing infection in high income countries, the *emm* type can be inferred from CRISPR analysis, which can further discriminate isolates sharing the same *emm* type.

## Introduction

Clustered regularly interspaced short palindromic repeats (CRISPR) are composed of serial spacer sequences flanked by repeats. CRISPR is now considered to be the prokaryotic adaptive immune system against foreign nucleic acid [1]. The mechanism involves two steps. The first step is an immunization. Prokaryotes can acquire small fragments from invading sequences, including phages or plasmids, to become new spacers in CRISPR. The second step is immunity. CRISPR can be transcribed from a leader promoter into a long RNA, and further processed into small RNAs containing one spacer and a partial repeat. Mature small RNAs, together with serial CRISPR-associated proteins (Cas), can recognize and degrade invading sequences complementary to spacer sequences. Therefore, CRISPR is like molecular “vaccination cards”, recording bacteria-virus interactions in spacer-repeat units [2], and providing adaptive immunity against foreign nucleic acids.

Since CRISPR is highly polymorphic, it has been used for bacterial typing. In *Mycobacterium tuberculosis*, 43 spacer sequences are used to differentiate epidemic clones, which is named spoligotyping [3]. In *Escherichia coli*, CRISPR is congruent with the evolutionary divergence of shiga toxin-producing *E*. *coli*, and has been used to detect hemorrhagic *E*. *coli* [4]. CRISPR typing also has good discriminatory powers in *Salmonella*, *Lactobacillus buchneri*, *Campylobacter jejuni*, *Propionibacterium acnes*, and *Erwinia amylovora* [5].

Group A streptococcus (GAS, *Streptococcus pyogenes*) is a Gram positive coccus. The diseases caused by GAS are varied, including pharyngitis, necrotizing fasciitis, and streptococcal toxic shock syndrome [6]. Traditionally, the serotype of GAS is determined by the surface virulence factor, M protein. However, this is limited by the availability of M typing sera. In recent years, sequencing of the *emm* gene, which encodes the M protein, has largely replaced the serotyping of the M protein [7]. The *emm* sequence typing is focused on the highly variable region of M protein. Two hundred and twenty three different *emm* types have been identified as of now, worldwide [8]. The prevalence rate of *emm* types is different among different regions, but the *emm*1 type has been the most common type since the 1980s, followed by *emm*12, *emm*28, *emm*3, and *emm*4 [9]. These were the most prevalent *emm* types in high-income countries. In Asia, *emm*1, *emm*4, and *emm*12 types are the most common types [9], and these types account for 79~89% of the isolates from scarlet fever and pediatric infections in central and southern Taiwan [10, 11].

In GAS, there are two CRISPR loci, which are named CRISPR01 and CRISPR02 [12]. Each CRISPR locus has its own *cas* cassette and CRISPR array. Experimental data demonstrate that the CRISPR01 locus of GAS strain SF370 can digest the invading nucleic acid, whereas the function of the CRISPR02 locus remains unclear [13]. The spacer contents and number of spacers are also associated with erythromycin susceptibility in *emm*12, *emm*75, and *emm*92 strains [14]. The aim of this study was to analyze the association between CRISPR and *emm* types in GAS, and we found that the *emm* type and spacer content of two CRISPR loci were associated.

## Materials and Methods

### Bacterial characterization

A total of 151 GAS isolates, including strain A20 (with a complete genome sequence) [15], were collected from National Cheng Kung University Hospital in southern Taiwan during 1994–2008 (S1 Table). Strain characterization, including identification, PFGE, and *emm* typing, was described in previous work [14]. A total of 170 non-redundant GAS strains with incomplete genome sequences and 11 strains with complete genome sequences were obtained from GenBank in the National Center for Biotechnology Information. The accession number and strain information of incomplete- and completely-sequenced strains are listed in S2 and S3 Tables, respectively.

### Determination of *emm* type

PCR amplification and sequencing of *emm* genes were performed as in previous descriptions [8]. To determine the *emm* type of strains with complete or incomplete genome sequences, the primer sequence of emm-Seq2 (tattcgcttagaaaattaaaaacagg) and emm-2 (gcaagttcttcagcttgttt) were used to search against GAS contigs by “somewhat similar sequences BLASTN” with default parameters. The targeted sequences from the emm-Seq2 to emm-2 region were used to search against the *emm* type-specific CDC database (http://www2a.cdc.gov/ncidod/biotech/strepblast.asp) to determine the *emm* type.

### Determination of *cas* cassette and CRISPR array in two CRISPR loci

The spacer contents of CRISPR01 and CRISPR02 were determined as described [14]. Briefly, CRISPR01 was amplified with primer CRISPR1-3 (cggtacaattcttgtgctcgaa) and CRISPR1-4 (tcaatggcgtttaacttgatgg) and sequenced with CRISPR1-1 (tgagaaacccgaagtgaa). CRISPR02 was amplified with primer CRISPR2-1 (tctgtgacacccgcagaattt) and CRISPR2-2 (aaaccagccccgtaacctaaa) and sequenced with CRISPR2-1. The spacer and repeat sequences were determined with the CRISPRtionary tool and CRISPR finder tool [16]. The presence of *cas* cassettes was determined by polymerase chain reaction in a previous study [14].

*In silico* analysis was used to determine the presence of CRISPR in strains with complete or incomplete genome sequences. The *cas* cassettes from CRISPR01 and CRISPR02 in MGAS9429 were used to search against the complete or incomplete genome sequences by megaBLASTN with default parameters. The contigs with *cas* cassettes were chosen. The presence of a CRISPR array in contigs with *cas* cassettes was identified by the CRISPR recognition tool and CRISPRFinder [17, 18]. The spacers in CRISPR01 and CRISPR02 loci were further identified by the CRISPRtionary tool [16]. Each unique spacer was designated with a specific numeral code.

### Estimation of diversity index and grouping comparison coefficients

Simpson’s index of diversity was used to measure the discriminatory ability of different typing methods [19]. This index indicates the probability that two strains which were randomly selected from a population belong to different types. The confidence interval (CI) and *p* value of Simpson’s index were calculated according to a previous study [20]. To compare different typing methods, the Adjusted Wallace coefficient was used to describe the directional relationship of congruence between two typing methods [21]. A higher score indicates two different typing methods have higher congruence. The CI of the adjusted Wallace coefficient was estimated as described previously [22]. The analysis was performed at Comparing Partitions (http://darwin.phyloviz.net/ComparingPartitions/index.php?link=Home).

### Statistical analysis

The Fisher's exact test was performed in SPSS software, version 17.0 (SPSS Inc., Chicago, IL, USA). A *p* value lower than 0.05 was considered to indicate statistical significance.

## Results

### Presence of CRISPR in GAS strains

A total of 182 non-redundant *emm*1, *emm*3, *emm*4, *emm*12, and *emm*28 strains with complete or incomplete genome sequences from NCBI were collected. Since these strains were not isolated in Taiwan (except *emm*1 strain A20, having a complete genome sequence), they were designated as “foreign” strains. To increase the strain collection, a total of 150 local strains, including 34, 49, and 67 strains of *emm*1, *emm*4, and *emm*12 types, respectively, were also included, which were designated as “local” strains.

Among 332 strains, all strains had at least one *cas* cassette or CRISPR array in the CRISPR01 or CRISPR02 loci (Table 1). The prevalence of a CRISPR array in the CRISPR01 locus was significantly less than in the CRISPR02 locus, while having a *cas* cassette in the CRISPR01 locus was more common than in the CRISPR02 locus (Table 1, *p* <0.001). All of the *emm*1, *emm*12, and *emm*28 strains had two intact CRISPR loci, including CRISPR array and *cas* cassette, except one *emm*1 strain (DSM 20565, S2 Table). *emm*3 and *emm*4 strains only had a *cas* cassette in the CRISPR01 locus. *emm*4 strains had a *cas* cassette and CRISPR array in the CRISPR02 locus, whereas *emm*3 strains did not have a CRISPR02 locus (Table 1). Thus, strains having these highly prevalent *emm* types had at least one *cas* cassette or CRISPR array.

**Table 1:** The prevalence of *cas* cassettes and CRISPR arrays in different *emm* types and regions of isolation.

			CRISPR01 locus			CRISPR02 locus		
*emm* type	Isolation region	No. of isolates	Intact locus	*cas*	CRISPR array	Intact locus	*cas*	CRISPR array
1	Foreign	54	53	54	53	54	54	54
	Local	35	35	35	35	35	35	35
3	Foreign	54	0	54	0	0	0	0
4	Foreign	2	0	2	0	2	2	2
	Local	49	0	49	0	49	49	49
12	Foreign	66	66	66	66	66	66	66
	Local	67	67	67	67	67	67	67
28	Foreign	5	5	5	5	5	5	5
Total (%)		332 (100%)	226 (68.1%, 226/332)	332 (100%, 332/332)	226 (68.1%, 226/332)	278 (83.7%, 278/332)	278 (83.7%, 278/332)	278 (83.7%, 278/332)

### Analysis of the spacer contents in CRISPR01 and CRISPR02 loci

A total of 14 and 21 unique spacer sequences were found in CRISPR01 and CRISPR02 loci, respectively, from all strains. In the CRISPR01 locus, 92.9% (13/14) of spacers were only found in one *emm* type, and 90.5% (19/21) of CRISPR02 spacers were specifically found in one *emm* type (S4 Table), suggesting that most CRISPR spacers were *emm* type-specific.

To further analyze the association between CRISPR and *emm* types, specific spacer contents were defined as a CRISPR type. In addition, the “CRISPRa type” was further defined as the combination of spacer contents from two CRISPR loci. Thus, based on spacer contents, there were 3 CRISPR types in one strain. Different numeral codes were used to represent different CRISPR types (Table 2). All CRISPR types were *emm* type-specific, except for CRISPR01 type 37, which corresponds to all isolates with a *cas* cassette but no CRISPR array in the CRISPR01 locus.

**Table 2:** The distribution of *emm* type, CRISPR01, CRISPR02, and CRISPRa type among local and foreign GAS isolates.

	CRISPR01		CRISPR02			No. of isolates		
*emm* type^#^	Type	Spacer content*	Type	Spacer content*	CRISPRa type	Foreign	Local	Total
1 (89)	7	-18-19-3-20-21-22-	8	-213-214-215-205-	34		1	1
	8	-23-21-	8	-213-214-215-205-	35	1		1
	9	-23-23-21-	8	-213-214-215-205-	36	1		1
	10	-23-23-3-21-	8	-213-214-215-205-	37		2	2
	11	-23-3-	8	-213-214-215-205-	38		12	12
	12	-23-3-21-	8	-213-214-215-205-	39	43	18	61
	13	-23-3-21-21-	8	-213-214-215-205-	40	1		1
	14	-23-77-21-	8	-213-214-215-205-	41	4		4
	19	-3-21-	8	-213-214-215-205-	47	2	2	4
	25	-44-19-3-20-21-22-	7	-213-214-215-	54	1		1
	37	*cas*+ CRISPR NO	8	-213-214-215-205-	73	1		1
3 (54)	37	*cas*+ CRISPR NO	55	No *cas*	80	54		54
4 (51)	37	*cas*+ CRISPR NO	12	-226-227-228-	75		1	1
	37	*cas*+ CRISPR NO	13	-226-227-228-229-230-	76	2	38	40
	37	*cas*+ CRISPR NO	14	-226-227-229-230-	77		5	5
	37	*cas*+ CRISPR NO	15	-226-228-229-230-	78		4	4
	37	*cas*+ CRISPR NO	16	-227-230-	79		1	1
12 (133)	1	-16-	42	-208-224-225-209-210-211-212-	1	1		1
	2	-17-	19	-237-208-224-225-209-211-212-	2		2	2
	2	-17-	42	-208-224-225-209-210-211-212-	3	1		1
	3	-16-16-17-	29	-250-251-252-208-224-225-209-210-211-212-	4	1		1
	4	-16-17-	1	-208-	5	1		1
	4	-16-17-	4	-210-211-212-	6	1	1	2
	4	-16-17-	19	-237-208-224-225-209-211-212-	7		3	3
	4	-16-17-	21	-250-210-211-212-	8	1		1
	4	-16-17-	22	-250-251-212-	9	1		1
	4	-16-17-	23	-250-251-225-209-210-	10	1		1
	4	-16-17-	24	-250-251-225-209-210-211-212-	11	1		1
	4	-16-17-	25	-250-251-252-225-209-210-211-212-	12	1		1
	4	-16-17-	26	-250-251-252-208-211-212-	13	5		5
	4	-16-17-	27	-250-251-252-208-224-211-212-	14	1		1
	4	-16-17-	28	-250-251-252-208-224-224-224-224-224-225-209-210-	15	1		1
	4	-16-17-	29	-250-251-252-208-224-225-209-210-211-212-	16	18		18
	4	-16-17-	30	-250-251-252-208-224-225-209-224-225-209-210-211-212-	17	1		1
	4	-16-17-	31	-250-251-252-208-224-289-209-210-211-212-	18	1		1
	4	-16-17-	32	-250-208-224-225-	19	1		1
	4	-16-17-	41	-208-224-224-210-211-212-	20	1		1
	4	-16-17-	42	-208-224-225-209-210-211-212-	21	20	9	29
	4	-16-17-	43	-208-224-225-209-210-212-	22	1		1
	4	-16-17-	44	-208-224-209-210-211-212-	23	1		1
	4	-16-17-	45	-208-209-210-	24	1		1
	4	-16-17-	46	-208-209-210-211-212-	25	1	10	11
	4	-16-17-	47	-208-209-211-212-	26	1		1
	4	-16-17-	48	-208-209-212-	27	1		1
	4	-16-17-	49	-209-210-211-212-	28		23	23
	4	-16-17-	50	-209-210-212-	29		17	17
	4	-16-17-	51	-209-211-212-	30		1	1
	4	-16-17-	52	-209-212-	31		1	1
	5	-16-17-17-	42	-208-224-225-209-210-211-212-	32	1		1
28 (5)	23	-36-37-17-28-	2	-229-	51	1		1
	23	-36-37-17-28-	17	-229-230-	52	4		4
					Total	181	151	332

* Each numeral code indicates a specific spacer. The “-” indicates the repeat sequence. “No *cas*” indicates that there is no *cas* cassette. “*cas*+ CRISPR NO” indicates the strain had a *cas* cassette, but no CRISPR array. The leader sequences are located at the left side of each spacer content, which are not shown in this table.

^#^ The number in parentheses indicates the total strain numbers in a specific *emm* type.

### Association between *emm* and CRISPR

CRISPR01 type 12 and CRISPR02 type 8 accounted for 68.5% (61/89) and 98.9% (88/89) of all *emm*1 strains, respectively (Table 2). CRISPR01 type 23 and CRISPR02 type 17 accounted for 100% (5/5) and 80% (4/5) of all *emm*28 strains, respectively (Table 2). In *emm*12, 95.5% (127/133) of the strains had CRISPR01 type 4. Although *emm*12 strains had diverse CRISPR02 loci, spacer No.212 from CRISPR02 was found in 96.2% of all *emm*12 isolates (128 of 133, Table 2).

All *emm*3 strains only had a *cas* cassette in the CRISPR01 locus. All *emm*4 strains had a *cas* cassette in the CRISPR01 locus, and had a CRISPR array and *cas* cassette in the CRISPR02 locus (Table 1). In *emm*4 strains, 78.4% (40/51) had CRISPR02 type 13 (Table 2). Since most *emm*4 strains were isolated from the local region, to rule out the possibility that CRISPR02 type 13 was the same clone, *Sma*l-digested PFGE typing was performed. The local *emm*4 strains with CRISPR02 type 13 had different PFGE types (data not shown), suggesting that the strains with a conserved CRISPR02 type were not closely related. Together, these results suggest that each *emm* type had their dominant CRISPR type.

**Fig 1:**
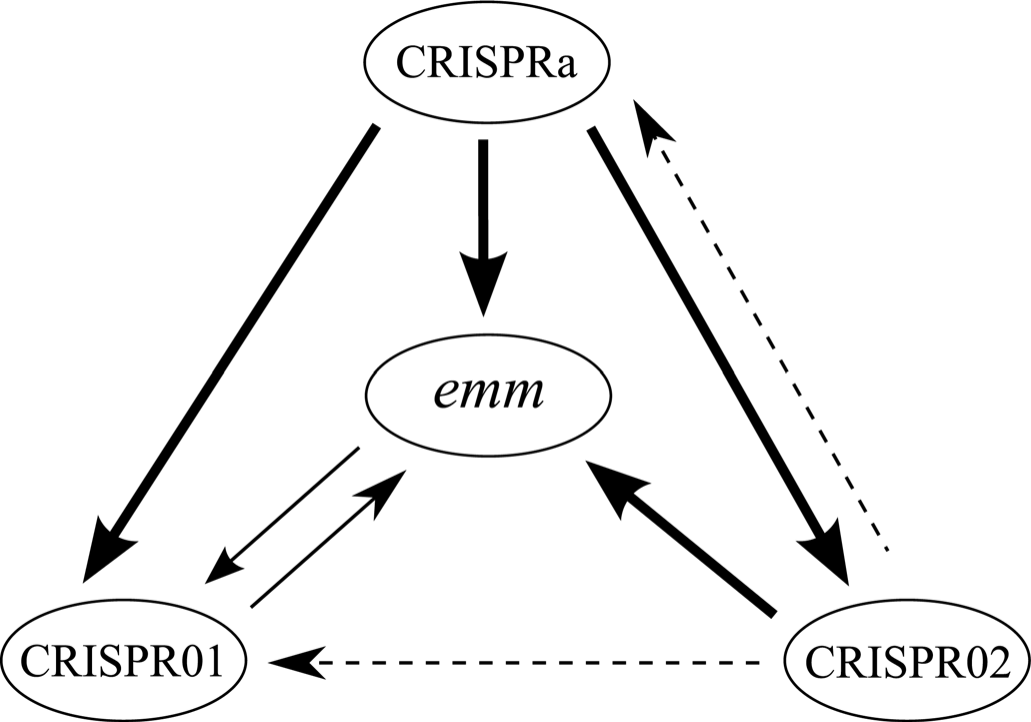
Correlations among *emm*, CRISPRa, CRISPR01, and CRISPR02 types. The congruence among different typing methods was estimated by Adjusted Wallace coefficients. The dash, thin, and thick arrows indicate Adjusted Wallace coefficients of 0.6~0.7, 0.7~0.9, and >0.9, respectively.

To compare the *emm* and the three CRISPR types, Simpson’s index of diversity was used. CRISPR01 and *emm* type presented a similar discriminatory power, according to the Simpson's ID (Table 3). However, CRISPR02 and CRISPRa types had a significantly higher discriminatory power when compared to *emm* type (Table 3). The association between *emm* and CRISPR01 types was supported by the adjusted Wallace method, which showed bidirectional congruence (Fig 1, the coefficients and 95% CI are listed in S5 Table), indicating that *emm* and CRISPR01 types were correlated. In addition, the adjusted Wallace coefficients from CRISPR02 and CRISPRa to *emm* type showed strong unidirectional congruence (Fig 1, the coefficients and 95% CI are listed in S5 Table), suggesting that the CRISPR02 and CRISPRa types can be used to infer the *emm* type among the highly prevalent *emm* types analyzed in this study.

To further demonstrate the robust relationship between CRISPR and *emm* types, the Simpson’s ID and adjusted Wallace coefficient between local and foreign strains were compared. Results showed that most values were not significantly different between local and foreign strains, except for the adjusted Wallace coefficients of *emm* to CRISPR02 and CRISPRa (Figs. A and B in S1 File). This suggests that the strong associations between CRISPS02/CRISPRa and *emm* type described for the whole dataset were robust and independent of region.

**Table 3:** Simpson’s index of diversity (ID) of *emm*, CRISPRa, CRISPR01, and CRISPR02 types.

			*p* values between Simpson's ID		
Typing method	No. of partitions	Simpson’s ID (95% CI)	*emm*	CRISPR01	CRISPR02
*emm*	5	0.720 (0.696–0.743)			
CRISPR01	17	0.718 (0.696–0.744)	0.923		
CRISPR02	37	0.869 (0.849–0.889)	<0.001	<0.001	
CRISPRa	51	0.906 (0.891–0.921)	<0.001	<0.001	<0.001

## Discussion

In this study, we have demonstrated that highly prevalent *emm* types of GAS strains had at least one *cas* cassette or CRISPR array, and more than 90% of spacers were *emm* type-specific. Based on Simpson’s index of diversity and adjusted Wallace coefficient, CRISPR01 and *emm* types were associated, and CRISPR02 showed strong unidirectional congruence to *emm* type, suggesting that CRISPR typing can be used as an alternative way to infer the *emm* type of GAS, at least among the highly prevalent *emm* types included in this study. Furthermore, because of the higher Simpson’s ID of CRISPR02 and CRISPRa, and the strong unidirectional congruence from CRISPR02 and CRISPRa to *emm* type, CRISPR typing can be also used to discriminate isolates of the same *emm* type. Further studies including more genetic lineages are required to compare the CRISPR typing with different typing methods, such as PFGE, multilocus sequence typing, or exotoxin profiles to demonstrate the value of CRISPR typing.

In the CRISPR01 locus of GAS, Hoe *et al* analyzed 30 *emm*1 strains collected from Texas. They found 9 different spacers [23]. Interestingly, 8 spacers of the Texas collection were comparable to our spacers in *emm*1 strains, further supporting that the spacers were *emm* type-specific.

Although CRISPR01 and *emm* type showed high congruence, all *emm*3 and *emm*4 strains had CRISPR01 type 37, indicating that the CRISPR01 type cannot distinguish between the *emm*3 and *emm*4 strains. Since *emm*4 strains had an intact CRISPR02 locus, when combined with the information from CRISPR02, *emm*3 and *emm*4 strains can be distinguished. In addition, the erythromycin resistance of *emm*12 strains has been associated with the spacer contents and number of spacers which were often found in the CRISPR02 type [14]. Therefore, CRISPR02 can not only distinguish the *emm* type, but also predict macrolide susceptibility, namely in *emm*12 strains.

Since combination of CRISPR01 and CRISPR02 types, or CRISPR02 type only can infer *emm* types, we propose that CRISPR typing can be performed in two alternative ways. First, the CRISPR01 and CRISPR02 arrays are amplified with primer CRISPR1-3 and CRISPR1-4, CRISPR2-1 and CRISPR2-2, respectively. The gel electrophoresis is performed to confirm the presence of two CRISPR arrays. The CRISPR01 array is further sequenced to determine its spacers. In our study, the sequence of CRISPR01 spacers combined with the PCR detection of the CRISPR02 array was sufficient to differentiate highly prevalent *emm* types, except one *emm*1 strain. Second, given the high value of the respective adjusted Wallace coefficient, CRISPR typing can be performed by amplifying and sequencing of CRISPR02 array only. It is not required to detect *cas* genes for inferring *emm* type. Furthermore, since the number of spacers was negatively associated with erythromycin susceptibility [14], the size of the CRISPR02 array obtained by PCR and gel electrophoresis can be used to infer the erythromycin susceptibility in *emm*12 strains.

Since the CRISPR array results from the bacteria-phage interaction, and phage diversity is tremendous, CRISPR spacers should be geographically specific [24–26]. However, our study showed the association between *emm* and CRISPR was robust among different regions, suggesting the CRISPR of GAS is independent of region. Interestingly, when the human gut microbiome was analyzed, unrelated people from different countries shared ~22% of spacers [27]. The reasons leading to form “conserved spacers” are still unclear. Possibly these strains shared recent common ancestry, or the conserved pattern was due to the slow insertion and deletion of spacers [28]. Population genomic studies showed that the contemporary *emm*1 and *emm*12 GAS strains originated in the early twentieth century [29, 30], suggesting recent common ancestry might exist in GAS. The number of spacers in GAS was less than in other streptococci [12], indicating that acquiring new spacers in GAS is not as efficient as in other streptococci, which may be due to the slow insertion and deletion of spacers. Another possibility to explain the robust association between CRISPR and *emm* types may be that the patterns of prophage-encoded virulence factors are associated with *emm* type [31, 32]. Since CRISPR arrays are expected to be closely associated with prophage content, it is possible that the association between CRISPR and *emm* type is due to the association between *emm* type and phage content. Further studies are required to support these hypotheses.

Our results suggest that CRISPR typing is a valuable typing method for GAS, although several limitations should be acknowledged in this study. Since most *emm*4 strains were collected locally and all *emm*28 strains were foreign, more geographically diverse strains of these *emm* types are required to analyze the association between *emm* and CRISPR types. In addition, strains of other less common *emm* types should also be included to further analyze this association. The correlation between *emm* and CRISPR types might be underrated due to the methodology used to identify CRISPR. In *Listeria monocytogenes*, *cas* gene-independent CRISPR is found [33]. However in our experiments, only the contigs with a *cas* cassette were chosen to analyze the presence of a CRISPR array. Therefore, the strains with a CRISPR array but without a *cas* cassette were missed in our study. Furthermore, studies from Africa and the Pacific region reported a much higher diversity of *emm* types, without a clear dominance of particular types [9]. Additionally, *emm*5, *emm*6, and *emm*18 strains did not have a CRISPR01 locus [12], which would limit the application of CRISPR typing in several *emm* types.

In summary, CRISPR spacers were *emm* type-specific in highly prevalent GAS, suggesting that at least for the majority of isolates causing infection in high income countries, the *emm* type can be inferred from CRISPR typing, which can further discriminate isolates sharing the same *emm* type.

## Supporting Information

S1 File Comparison of Simpson’s ID (Figure A in S1 File) and adjusted Wallace coefficients (Figure B in S1 File) between local and foreign strains. (DOCX)

S1 Table The *emm* type, year of isolation, and CRISPR information of local strains used in this study. (DOCX)

S2 Table The *emm* type and CRISPR information of foreign strains with incomplete genomes used in this study. (DOCX)

S3 Table The *emm* type, accession number, and CRISPR information of strains with complete genomes used in this study (DOCX)

S4 Table Spacer sequences from CRISPR01 and CRISPR02 and their relationship to *emm* type (DOCX)

S5 Table Adjusted Wallace coefficients and jackknife pseudo-value 95% confident interval (CI) for the *emm*, CRISPRa, CRISPR01, and CRISPR02 types among all foreign and local strains. (DOCX)
