# Supplementary material for: Clustered Regularly Interspaced Short Palindromic Repeats Are emm Type-Specific in Highly Prevalent Group A Streptococci
Source: PLoS One. 2015 Dec 28;10(12):e0145223. doi: 10.1371/journal.pone.0145223 (PMC4692479; doi:10.1371/journal.pone.0145223)

**S1 File. Comparison of Simpson’s ID (Figure A) and adjusted Wallace coefficients (Figure B) between local and foreign strains.** The white and grey bars indicate the local and foreign strains, respectively. The error bars represent the 95% confidence interval. * indicates the *p* <0.05.


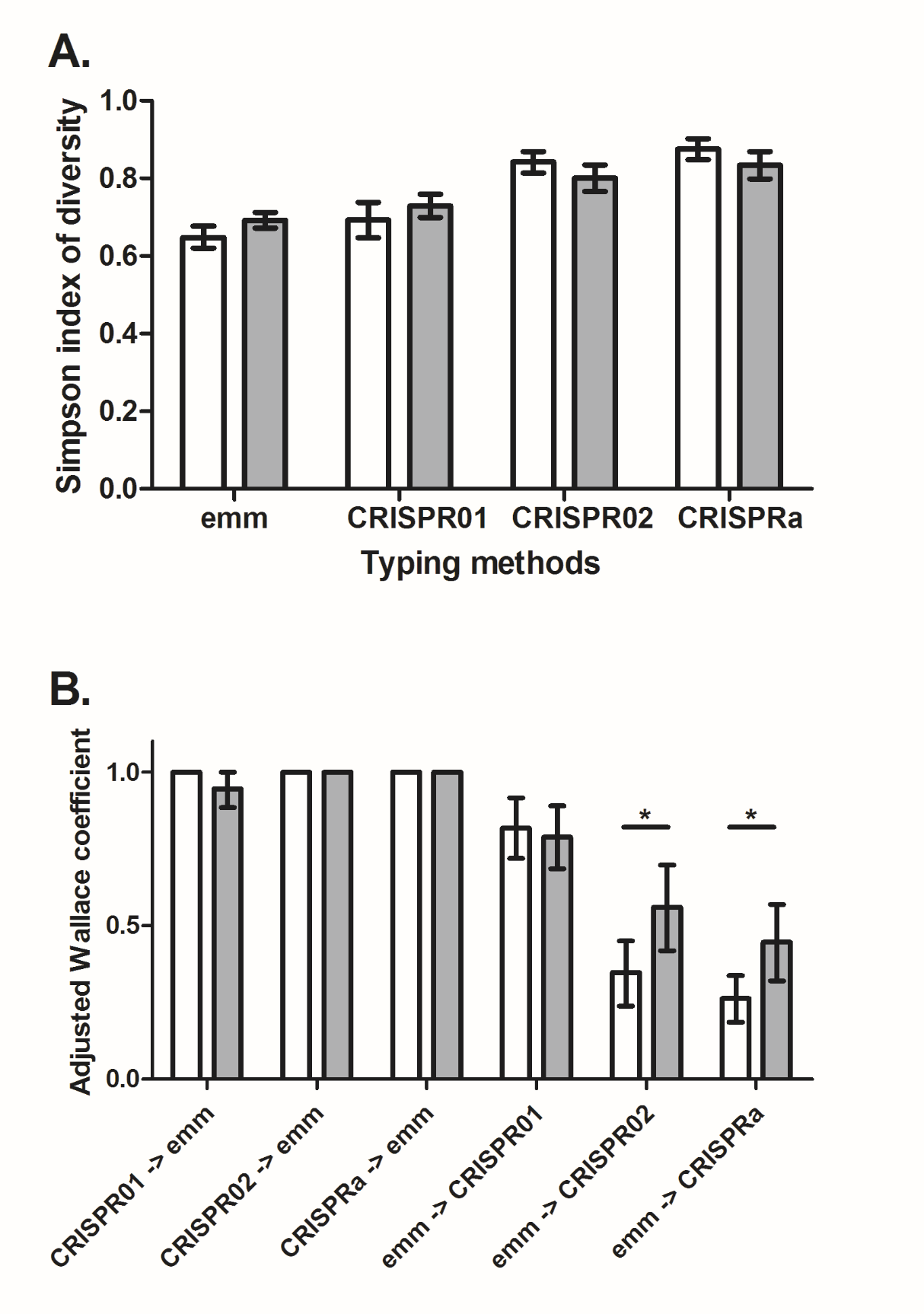

Supplement: S1 File — (DOCX) [file pone.0145223.s001.docx]
