# Supplementary material for: Clustered Regularly Interspaced Short Palindromic Repeats Are emm Type-Specific in Highly Prevalent Group A Streptococci
Source: PLoS One. 2015 Dec 28;10(12):e0145223. doi: 10.1371/journal.pone.0145223 (PMC4692479; doi:10.1371/journal.pone.0145223)
Supplement: S1 Table — (DOCX) [file pone.0145223.s002.docx]

**S1 Table.** The *emm* type, year of isolation, and CRISPR information of local strains used in this study

|  |  |  | CRISPR01 | |  | CRISPR02 | |  |
| --- | --- | --- | --- | --- | --- | --- | --- | --- |
| Strain | Year of isolation | *emm* type | Type | Spacer content * |  | Type | Spacer content * | CRISPRa type |
| 7 | 1994 | 1 | 12 | -23-3-21- |  | 8 | -213-214-215-205- | 39 |
| 22 | 1995 | 1 | 7 | -18-19-3-20-21-22- |  | 8 | -213-214-215-205- | 34 |
| 81 | 1994 | 1 | 12 | -23-3-21- |  | 8 | -213-214-215-205- | 39 |
| 152 | 1997 | 4 | 37 | cas+ CRISPR No |  | 13 | -226-227-228-229-230- | 76 |
| 155 | 1997 | 4 | 37 | cas+ CRISPR No |  | 13 | -226-227-228-229-230- | 76 |
| 156 | 1997 | 4 | 37 | cas+ CRISPR No |  | 13 | -226-227-228-229-230- | 76 |
| 160 | 1997 | 4 | 37 | cas+ CRISPR No |  | 13 | -226-227-228-229-230- | 76 |
| 161 | 1997 | 4 | 37 | cas+ CRISPR No |  | 13 | -226-227-228-229-230- | 76 |
| 162 | 1997 | 4 | 37 | cas+ CRISPR No |  | 13 | -226-227-228-229-230- | 76 |
| 164 | 1997 | 1 | 12 | -23-3-21- |  | 8 | -213-214-215-205- | 39 |
| 165 | 1997 | 1 | 12 | -23-3-21- |  | 8 | -213-214-215-205- | 39 |
| 170 | 1997 | 4 | 37 | cas+ CRISPR No |  | 13 | -226-227-228-229-230- | 76 |
| 172 | 1997 | 4 | 37 | cas+ CRISPR No |  | 13 | -226-227-228-229-230- | 76 |
| 173 | 1997 | 4 | 37 | cas+ CRISPR No |  | 13 | -226-227-228-229-230- | 76 |
| 175 | 1997 | 4 | 37 | cas+ CRISPR No |  | 13 | -226-227-228-229-230- | 76 |
| 176 | 1997 | 4 | 37 | cas+ CRISPR No |  | 13 | -226-227-228-229-230- | 76 |
| 178 | 1997 | 1 | 12 | -23-3-21- |  | 8 | -213-214-215-205- | 39 |
| 179 | 1997 | 12 | 4 | -16-17- |  | 50 | -209-210-212- | 29 |
| 201 | 1997 | 4 | 37 | cas+ CRISPR No |  | 13 | -226-227-228-229-230- | 76 |
| 204 | 1997 | 4 | 37 | cas+ CRISPR No |  | 13 | -226-227-228-229-230- | 76 |
| 205 | 1997 | 4 | 37 | cas+ CRISPR No |  | 15 | -226-228-229-230- | 78 |
| 208 | 1997 | 4 | 37 | cas+ CRISPR No |  | 14 | -226-227-229-230- | 77 |
| 210 | 1997 | 4 | 37 | cas+ CRISPR No |  | 14 | -226-227-229-230- | 77 |
| 211 | 1997 | 4 | 37 | cas+ CRISPR No |  | 13 | -226-227-228-229-230- | 76 |
| 215 | 1997 | 12 | 4 | -16-17- |  | 42 | -208-224-225-209-210-211-212- | 21 |
| 216 | 1997 | 12 | 4 | -16-17- |  | 42 | -208-224-225-209-210-211-212- | 21 |
| 217 | 1998 | 4 | 37 | cas+ CRISPR No |  | 13 | -226-227-228-229-230- | 76 |
| 218 | 1997 | 4 | 37 | cas+ CRISPR No |  | 14 | -226-227-229-230- | 77 |
| 220 | 1997 | 4 | 37 | cas+ CRISPR No |  | 13 | -226-227-228-229-230- | 76 |
| 221 | 1997 | 4 | 37 | cas+ CRISPR No |  | 13 | -226-227-228-229-230- | 76 |
| 227 | 1997 | 4 | 37 | cas+ CRISPR No |  | 13 | -226-227-228-229-230- | 76 |
| 229 | 1997 | 4 | 37 | cas+ CRISPR No |  | 14 | -226-227-229-230- | 77 |
| 230 | 1997 | 12 | 4 | -16-17- |  | 50 | -209-210-212- | 29 |
| 232 | 1997 | 12 | 4 | -16-17- |  | 49 | -209-210-211-212- | 28 |
| 237 | 1997 | 4 | 37 | cas+ CRISPR No |  | 12 | -226-227-228- | 75 |
| 239 | 1997 | 4 | 37 | cas+ CRISPR No |  | 13 | -226-227-228-229-230- | 76 |
| 246 | 1997 | 4 | 37 | cas+ CRISPR No |  | 13 | -226-227-228-229-230- | 76 |
| 248 | 1998 | 12 | 4 | -16-17- |  | 42 | -208-224-225-209-210-211-212- | 21 |
| 251 | 1998 | 12 | 4 | -16-17- |  | 50 | -209-210-212- | 29 |
| 252 | 1998 | 12 | 4 | -16-17- |  | 49 | -209-210-211-212- | 28 |
| 261 | 1998 | 12 | 4 | -16-17- |  | 50 | -209-210-212- | 29 |
| 263 | 1998 | 4 | 37 | cas+ CRISPR No |  | 13 | -226-227-228-229-230- | 76 |
| 264 | 1998 | 4 | 37 | cas+ CRISPR No |  | 13 | -226-227-228-229-230- | 76 |
| 265 | 1998 | 4 | 37 | cas+ CRISPR No |  | 13 | -226-227-228-229-230- | 76 |
| 268 | 1998 | 4 | 37 | cas+ CRISPR No |  | 14 | -226-227-229-230- | 77 |
| 270 | 1998 | 4 | 37 | cas+ CRISPR No |  | 13 | -226-227-228-229-230- | 76 |
| 271 | 1998 | 12 | 4 | -16-17- |  | 49 | -209-210-211-212- | 28 |
| 275 | 1998 | 12 | 4 | -16-17- |  | 49 | -209-210-211-212- | 28 |
| 501 | 1998 | 1 | 12 | -23-3-21- |  | 8 | -213-214-215-205- | 39 |
| 502 | 1998 | 4 | 37 | cas+ CRISPR No |  | 13 | -226-227-228-229-230- | 76 |
| 504 | 1999 | 12 | 4 | -16-17- |  | 49 | -209-210-211-212- | 28 |
| 506 | 1999 | 12 | 4 | -16-17- |  | 49 | -209-210-211-212- | 28 |
| 508 | 1999 | 4 | 37 | cas+ CRISPR No |  | 13 | -226-227-228-229-230- | 76 |
| 511 | 1999 | 12 | 4 | -16-17- |  | 50 | -209-210-212- | 29 |
| 513 | 1999 | 12 | 4 | -16-17- |  | 42 | -208-224-225-209-210-211-212- | 21 |
| 518 | 1999 | 12 | 4 | -16-17- |  | 51 | -209-211-212- | 30 |
| 519 | 1999 | 4 | 37 | cas+ CRISPR No |  | 13 | -226-227-228-229-230- | 76 |
| 537 | 1999 | 4 | 37 | cas+ CRISPR No |  | 13 | -226-227-228-229-230- | 76 |
| 539 | 1999 | 12 | 4 | -16-17- |  | 50 | -209-210-212- | 29 |
| 541 | 1999 | 12 | 4 | -16-17- |  | 49 | -209-210-211-212- | 28 |
| 544 | 1999 | 12 | 4 | -16-17- |  | 49 | -209-210-211-212- | 28 |
| 545 | 1999 | 12 | 4 | -16-17- |  | 50 | -209-210-212- | 29 |
| 546 | 1999 | 12 | 4 | -16-17- |  | 50 | -209-210-212- | 29 |
| 547 | 2000 | 12 | 4 | -16-17- |  | 50 | -209-210-212- | 29 |
| 548 | 2000 | 12 | 4 | -16-17- |  | 49 | -209-210-211-212- | 28 |
| 550 | 2000 | 12 | 4 | -16-17- |  | 46 | -208-209-210-211-212- | 25 |
| 551 | 2000 | 12 | 4 | -16-17- |  | 50 | -209-210-212- | 29 |
| 552 | 2000 | 4 | 37 | cas+ CRISPR No |  | 16 | -227-230- | 79 |
| 553 | 2000 | 4 | 37 | cas+ CRISPR No |  | 13 | -226-227-228-229-230- | 76 |
| 555 | 2000 | 12 | 4 | -16-17- |  | 50 | -209-210-212- | 29 |
| 556 | 2000 | 12 | 4 | -16-17- |  | 50 | -209-210-212- | 29 |
| 557 | 2000 | 12 | 4 | -16-17- |  | 50 | -209-210-212- | 29 |
| 558 | 2000 | 12 | 4 | -16-17- |  | 50 | -209-210-212- | 29 |
| 560 | 2000 | 12 | 4 | -16-17- |  | 49 | -209-210-211-212- | 28 |
| 563 | 2000 | 12 | 2 | -17- |  | 19 | -237-208-224-225-209-211-212- | 2 |
| 564 | 2000 | 12 | 4 | -16-17- |  | 49 | -209-210-211-212- | 28 |
| 565 | 2000 | 12 | 4 | -16-17- |  | 49 | -209-210-211-212- | 28 |
| 566 | 2000 | 12 | 4 | -16-17- |  | 49 | -209-210-211-212- | 28 |
| 567 | 2000 | 12 | 4 | -16-17- |  | 19 | -237-208-224-225-209-211-212- | 7 |
| 568 | 2000 | 12 | 4 | -16-17- |  | 19 | -237-208-224-225-209-211-212- | 7 |
| 569 | 2000 | 12 | 4 | -16-17- |  | 19 | -237-208-224-225-209-211-212- | 7 |
| 573 | 2000 | 12 | 4 | -16-17- |  | 52 | -209-212- | 31 |
| 574 | 2000 | 12 | 4 | -16-17- |  | 46 | -208-209-210-211-212- | 25 |
| 576 | 2000 | 12 | 4 | -16-17- |  | 49 | -209-210-211-212- | 28 |
| 578 | 2000 | 12 | 4 | -16-17- |  | 49 | -209-210-211-212- | 28 |
| 601 | 2000 | 12 | 4 | -16-17- |  | 49 | -209-210-211-212- | 28 |
| 603 | 2000 | 12 | 4 | -16-17- |  | 49 | -209-210-211-212- | 28 |
| 604 | 2000 | 12 | 4 | -16-17- |  | 42 | -208-224-225-209-210-211-212- | 21 |
| 605 | 2000 | 4 | 37 | cas+ CRISPR No |  | 13 | -226-227-228-229-230- | 76 |
| 609 | 2000 | 12 | 4 | -16-17- |  | 46 | -208-209-210-211-212- | 25 |
| 615 | 2000 | 4 | 37 | cas+ CRISPR No |  | 15 | -226-228-229-230- | 78 |
| 619 | 2000 | 4 | 37 | cas+ CRISPR No |  | 13 | -226-227-228-229-230- | 76 |
| 620 | 2000 | 4 | 37 | cas+ CRISPR No |  | 13 | -226-227-228-229-230- | 76 |
| 621 | 2001 | 12 | 4 | -16-17- |  | 46 | -208-209-210-211-212- | 25 |
| 623 | 2001 | 12 | 4 | -16-17- |  | 49 | -209-210-211-212- | 28 |
| 626 | 2001 | 12 | 4 | -16-17- |  | 46 | -208-209-210-211-212- | 25 |
| 630 | 2001 | 12 | 2 | -17- |  | 19 | -237-208-224-225-209-211-212- | 2 |
| 631 | 2001 | 4 | 37 | cas+ CRISPR No |  | 13 | -226-227-228-229-230- | 76 |
| 632 | 2001 | 12 | 4 | -16-17- |  | 49 | -209-210-211-212- | 28 |
| 633 | 2001 | 4 | 37 | cas+ CRISPR No |  | 13 | -226-227-228-229-230- | 76 |
| 634 | 2001 | 1 | 19 | -3-21- |  | 8 | -213-214-215-205- | 47 |
| 635 | 2001 | 12 | 4 | -16-17- |  | 46 | -208-209-210-211-212- | 25 |
| 640 | 2001 | 12 | 4 | -16-17- |  | 50 | -209-210-212- | 29 |
| 642 | 2001 | 12 | 4 | -16-17- |  | 50 | -209-210-212- | 29 |
| 643 | 2002 | 4 | 37 | cas+ CRISPR No |  | 15 | -226-228-229-230- | 78 |
| 645 | 2002 | 4 | 37 | cas+ CRISPR No |  | 13 | -226-227-228-229-230- | 76 |
| 646 | 2002 | 1 | 12 | -23-3-21- |  | 8 | -213-214-215-205- | 39 |
| 647 | 2002 | 12 | 4 | -16-17- |  | 42 | -208-224-225-209-210-211-212- | 21 |
| 653 | 2002 | 1 | 12 | -23-3-21- |  | 8 | -213-214-215-205- | 39 |
| 654 | 2002 | 1 | 12 | -23-3-21- |  | 8 | -213-214-215-205- | 39 |
| 658 | 2002 | 12 | 4 | -16-17- |  | 49 | -209-210-211-212- | 28 |
| 659 | 2002 | 12 | 4 | -16-17- |  | 49 | -209-210-211-212- | 28 |
| 662 | 2002 | 12 | 4 | -16-17- |  | 50 | -209-210-212- | 29 |
| 664 | 2002 | 1 | 12 | -23-3-21- |  | 8 | -213-214-215-205- | 39 |
| 667 | 2002 | 1 | 12 | -23-3-21- |  | 8 | -213-214-215-205- | 39 |
| 669 | 2002 | 1 | 11 | -23-3- |  | 8 | -213-214-215-205- | 38 |
| 679 | 2002 | 12 | 4 | -16-17- |  | 46 | -208-209-210-211-212- | 25 |
| 706 | 2002 | 1 | 11 | -23-3- |  | 8 | -213-214-215-205- | 38 |
| 719 | 2003 | 12 | 4 | -16-17- |  | 46 | -208-209-210-211-212- | 25 |
| 723 | 2003 | 12 | 4 | -16-17- |  | 46 | -208-209-210-211-212- | 25 |
| 724 | 2003 | 1 | 11 | -23-3- |  | 8 | -213-214-215-205- | 38 |
| 728 | 2003 | 1 | 12 | -23-3-21- |  | 8 | -213-214-215-205- | 39 |
| 730 | 2003 | 12 | 4 | -16-17- |  | 46 | -208-209-210-211-212- | 25 |
| 731 | 2003 | 1 | 12 | -23-3-21- |  | 8 | -213-214-215-205- | 39 |
| 733 | 2003 | 12 | 4 | -16-17- |  | 4 | -210-211-212- | 6 |
| 734 | 2003 | 12 | 4 | -16-17- |  | 49 | -209-210-211-212- | 28 |
| 737 | 2003 | 1 | 11 | -23-3- |  | 8 | -213-214-215-205- | 38 |
| 738 | 2003 | 1 | 12 | -23-3-21- |  | 8 | -213-214-215-205- | 39 |
| 740 | 2003 | 12 | 4 | -16-17- |  | 42 | -208-224-225-209-210-211-212- | 21 |
| 742 | 2003 | 1 | 11 | -23-3- |  | 8 | -213-214-215-205- | 38 |
| 743 | 2003 | 4 | 37 | cas+ CRISPR No |  | 15 | -226-228-229-230- | 78 |
| 746 | 2003 | 1 | 11 | -23-3- |  | 8 | -213-214-215-205- | 38 |
| 747 | 2003 | 12 | 4 | -16-17- |  | 42 | -208-224-225-209-210-211-212- | 21 |
| 749 | 2003 | 1 | 12 | -23-3-21- |  | 8 | -213-214-215-205- | 39 |
| 754 | 2003 | 12 | 4 | -16-17- |  | 49 | -209-210-211-212- | 28 |
| 759 | 2003 | 12 | 4 | -16-17- |  | 42 | -208-224-225-209-210-211-212- | 21 |
| 762 | 2003 | 1 | 11 | -23-3- |  | 8 | -213-214-215-205- | 38 |
| 773 | 2003 | 1 | 12 | -23-3-21- |  | 8 | -213-214-215-205- | 39 |
| 773 | 2003 | 1 | 12 | -23-3-21- |  | 8 | -213-214-215-205- | 39 |
| 779 | 2003 | 4 | 37 | cas+ CRISPR No |  | 13 | -226-227-228-229-230- | 76 |
| 802 | 2003 | 4 | 37 | cas+ CRISPR No |  | 13 | -226-227-228-229-230- | 76 |
| 804 | 2003 | 4 | 37 | cas+ CRISPR No |  | 13 | -226-227-228-229-230- | 76 |
| 810 | 2004 | 1 | 19 | -3-21- |  | 8 | -213-214-215-205- | 47 |
| 813 | 2004 | 1 | 11 | -23-3- |  | 8 | -213-214-215-205- | 38 |
| 817 | 2004 | 1 | 11 | -23-3- |  | 8 | -213-214-215-205- | 38 |
| 841 | 2004 | 1 | 11 | -23-3- |  | 8 | -213-214-215-205- | 38 |
| 843 | 2004 | 1 | 11 | -23-3- |  | 8 | -213-214-215-205- | 38 |
| 1104 | 2007 | 1 | 11 | -23-3- |  | 8 | -213-214-215-205- | 38 |
| 1115 | 2008 | 1 | 10 | -23-23-3-21- |  | 8 | -213-214-215-205- | 37 |
| 1117 | 2008 | 1 | 10 | -23-23-3-21- |  | 8 | -213-214-215-205- | 37 |
| A20 ^#^ | 1994 | 1 | 12 | -23-3-21- |  | 8 | -213-214-215-205- | 39 |

* Each Number indicates a specific spacer. The “-” indicates the repeat sequence. “No cas” indicates that there is no *cas* genes cassette. “cas+ CRISPR NO” indicates the strain had a *cas* cassette, but no CRISPR array. The lead sequences are located at the left side of each spacer content, which is not shown in this Table.

^#^ The complete genome of strain A20 was determined (accession No. NC_018936).
