# Supplementary material for: Clustered Regularly Interspaced Short Palindromic Repeats Are emm Type-Specific in Highly Prevalent Group A Streptococci
Source: PLoS One. 2015 Dec 28;10(12):e0145223. doi: 10.1371/journal.pone.0145223 (PMC4692479; doi:10.1371/journal.pone.0145223)
Supplement: S2 Table — (DOCX) [file pone.0145223.s003.docx]

**S2 Table.** The *emm* type and CRISPR information of foreign strains with incomplete genomes used in this study

|  |  |  | CRISPR01 |  |  |  | CRISPR02 |  |  |
| --- | --- | --- | --- | --- | --- | --- | --- | --- | --- |
| strain | *emm* type | Type | Spacer content * | Contig with CRISPR ^#^ |  | Type | Spacer content * | Contig with CRISPR ^#^ | CRISPRa type |
| ABC020004984 | 3 | 37 | cas+ CRISPR No | JJGE01000006.1 |  | 55 | No cas | ND | 80 |
| ABC020005405 | 12 | 4 | -16-17- | JJGD01000001.1 |  | 29 | -250-251-252-208-224-225-209-210-211-212- | JJGD01000001.1 | 16 |
| ABC020005716 | 1 | 12 | -23-3-21- | JJKE01000001.1 |  | 8 | -213-214-215-205- | JJKE01000001.1 | 39 |
| ABC020005773 | 3 | 37 | cas+ CRISPR No | JJKF01000002.1 |  | 55 | No cas | ND | 80 |
| ABC020005887 | 1 | 12 | -23-3-21- | JJHO01000003.1 |  | 8 | -213-214-215-205- | JJHO01000003.1 | 39 |
| ABC020006103 | 3 | 37 | cas+ CRISPR No | JJHN01000001.1 |  | 55 | No cas | ND | 80 |
| ABC020006298 | 3 | 37 | cas+ CRISPR No | JJGC01000002.1 |  | 55 | No cas | ND | 80 |
| ABC020006309 | 12 | 4 | -16-17- | JJGB01000001.1 |  | 26 | -250-251-252-208-211-212- | JJGB01000001.1 | 13 |
| ABC020006345 | 1 | 12 | -23-3-21- | JJKG01000001.1 |  | 8 | -213-214-215-205- | JJKG01000001.1 | 39 |
| ABC020006715 | 1 | 12 | -23-3-21- | JJKH01000001.1 |  | 8 | -213-214-215-205- | JJKH01000001.1 | 39 |
| ABC020007794 | 12 | 4 | -16-17- | JJGA01000001.1 |  | 29 | -250-251-252-208-224-225-209-210-211-212- | JJGA01000002.1 | 16 |
| ABC020009156 | 12 | 4 | -16-17- | JJFZ01000001.1 |  | 29 | -250-251-252-208-224-225-209-210-211-212- | JJFZ01000002.1 | 16 |
| ABC020010041 | 12 | 4 | -16-17- | JJFY01000004.1 |  | 29 | -250-251-252-208-224-225-209-210-211-212- | JJFY01000004.1 | 16 |
| ABC020013256 | 1 | 12 | -23-3-21- | JJKV01000001.1 |  | 8 | -213-214-215-205- | JJKV01000001.1 | 39 |
| ABC020013551 | 3 | 37 | cas+ CRISPR No | JJFX01000003.1 |  | 55 | No cas | ND | 80 |
| ABC020013952 | 12 | 4 | -16-17- | JJHM01000001.1 |  | 42 | -208-224-225-209-210-211-212- | JJHM01000002.1 | 21 |
| ABC020014529 | 12 | 4 | -16-17- | JJHL01000001.1 |  | 42 | -208-224-225-209-210-211-212- | JJHL01000001.1 | 21 |
| ABC020014690 | 12 | 4 | -16-17- | JJFW01000001.1 |  | 41 | -208-224-224-210-211-212- | JJFW01000002.1 | 20 |
| ABC020014764 | 1 | 12 | -23-3-21- | JJKI01000001.1 |  | 8 | -213-214-215-205- | JJKI01000001.1 | 39 |
| ABC020014897 | 3 | 37 | cas+ CRISPR No | JJHK01000004.1 |  | 55 | No cas | ND | 80 |
| ABC020014925 | 3 | 37 | cas+ CRISPR No | JJHJ01000004.1 |  | 55 | No cas | ND | 80 |
| ABC020015277 | 12 | 4 | -16-17- | JJFV01000001.1 |  | 32 | -250-208-224-225- | JJFV01000001.1 | 19 |
| ABC020015285 | 12 | 5 | -16-17-17- | JJFU01000001.1 |  | 42 | -208-224-225-209-210-211-212- | JJFU01000002.1 | 32 |
| ABC020015292 | 3 | 37 | cas+ CRISPR No | JJFT01000002.1 |  | 55 | No cas | ND | 80 |
| ABC020015294 | 1 | 12 | -23-3-21- | JJFS01000001.1 |  | 8 | -213-214-215-205- | JJFS01000001.1 | 39 |
| ABC020016937 | 12 | 4 | -16-17- | JJFR01000002.1 |  | 42 | -208-224-225-209-210-211-212- | JJFR01000002.1 | 21 |
| ABC020017280 | 12 | 4 | -16-17- | JJKW01000001.1 |  | 26 | -250-251-252-208-211-212- | JJKW01000001.1 | 13 |
| ABC020017526 | 3 | 37 | cas+ CRISPR No | JJHI01000001.1 |  | 55 | No cas | ND | 80 |
| ABC020017774 | 1 | 12 | -23-3-21- | JJKX01000001.1 |  | 8 | -213-214-215-205- | JJKX01000001.1 | 39 |
| ABC020021452 | 1 | 12 | -23-3-21- | JJKY01000001.1 |  | 8 | -213-214-215-205- | JJKY01000001.1 | 39 |
| ABC020025676 | 3 | 37 | cas+ CRISPR No | JJFQ01000002.1 |  | 55 | No cas | ND | 80 |
| ABC020026287 | 3 | 37 | cas+ CRISPR No | JJHH01000004.1 |  | 55 | No cas | ND | 80 |
| ABC020026799 | 3 | 37 | cas+ CRISPR No | JJHG01000001.1 |  | 55 | No cas | ND | 80 |
| ABC020026946 | 12 | 4 | -16-17- | JJFP01000002.1 |  | 45 | -208-209-210- | JJFP01000002.1 | 24 |
| ABC020029706 | 12 | 4 | -16-17- | JJIT01000002.1 |  | 29 | -250-251-252-208-224-225-209-210-211-212- | JJIT01000002.1 | 16 |
| ABC020029711 | 1 | 12 | -23-3-21- | JJHF01000002.1 |  | 8 | -213-214-215-205- | JJHF01000002.1 | 39 |
| ABC020029793 | 12 | 4 | -16-17- | JJHE01000001.1 |  | 42 | -208-224-225-209-210-211-212- | JJHE01000001.1 | 21 |
| ABC020030020 | 1 | 19 | -3-21- | JJKJ01000001.1 |  | 8 | -213-214-215-205- | JJKJ01000001.1 | 47 |
| ABC020030063 | 3 | 37 | cas+ CRISPR No | JJIC01000002.1 |  | 55 | No cas | ND | 80 |
| ABC020031290 | 12 | 4 | -16-17- | JJHC01000001.1 |  | 42 | -208-224-225-209-210-211-212- | JJHC01000001.1 | 21 |
| ABC020031898 | 12 | 4 | -16-17- | JJFO01000001.1 |  | 29 | -250-251-252-208-224-225-209-210-211-212- | JJFO01000002.1 | 16 |
| ABC020032057 | 1 | 8 | -23-21- | JJKK01000001.1 |  | 8 | -213-214-215-205- | JJKK01000001.1 | 35 |
| ABC020032182 | 3 | 37 | cas+ CRISPR No | JJHB01000001.1 |  | 55 | No cas | ND | 80 |
| ABC020032183 | 12 | 4 | -16-17- | JJFN01000001.1 |  | 23 | -250-251-225-209-210- | JJFN01000002.1 | 10 |
| ABC020032186 | 12 | 4 | -16-17- | JJIR01000001.1 |  | 29 | -250-251-252-208-224-225-209-210-211-212- | JJIR01000002.1 | 16 |
| ABC020032523 | 12 | 4 | -16-17- | JJHA01000001.1 |  | 29 | -250-251-252-208-224-225-209-210-211-212- | JJHA01000001.1 | 16 |
| ABC020032535 | 1 | 12 | -23-3-21- | JJKL01000001.1 |  | 8 | -213-214-215-205- | JJKL01000001.1 | 39 |
| ABC020033020 | 3 | 37 | cas+ CRISPR No | JJIB01000003.1 |  | 55 | No cas | ND | 80 |
| ABC020033193 | 12 | 4 | -16-17- | JJGZ01000001.1 |  | 21 | -250-210-211-212- | JJGZ01000001.1 | 8 |
| ABC020033233 | 12 | 4 | -16-17- | JJIS01000001.1 |  | 42 | -208-224-225-209-210-211-212- | JJIS01000002.1 | 21 |
| ABC020033799 | 12 | 4 | -16-17- | JJGY01000002.1 |  | 31 | -250-251-252-208-224-289-209-210-211-212- | JJGY01000002.1 | 18 |
| ABC020033919 | 12 | 4 | -16-17- | JJIQ01000001.1 |  | 29 | -250-251-252-208-224-225-209-210-211-212- | JJIQ01000001.1 | 16 |
| ABC020035427 | 12 | 4 | -16-17- | JJFM01000001.1 |  | 42 | -208-224-225-209-210-211-212- | JJFM01000002.1 | 21 |
| ABC020035446 | 12 | 4 | -16-17- | JJIP01000004.1 |  | 29 | -250-251-252-208-224-225-209-210-211-212- | JJIP01000004.1 | 16 |
| ABC020037542 | 12 | 4 | -16-17- | JJIO01000001.1 |  | 1 | -208- | JJIO01000001.1 | 5 |
| ABC020038544 | 12 | 4 | -16-17- | JJJG01000003.1 |  | 42 | -208-224-225-209-210-211-212- | JJJG01000004.1 | 21 |
| ABC020038545 | 1 | 12 | -23-3-21- | JJKM01000001.1 |  | 8 | -213-214-215-205- | JJKM01000001.1 | 39 |
| ABC020038558 | 3 | 37 | cas+ CRISPR No | JJGX01000002.1 |  | 55 | No cas | ND | 80 |
| ABC020039031 | 12 | 4 | -16-17- | JJIN01000001.1 |  | 42 | -208-224-225-209-210-211-212- | JJIN01000002.1 | 21 |
| ABC020039391 | 12 | 4 | -16-17- | JJGW01000001.1 |  | 26 | -250-251-252-208-211-212- | JJGW01000003.1 | 13 |
| ABC020040643 | 12 | 4 | -16-17- | JJJF01000002.1 |  | 29 | -250-251-252-208-224-225-209-210-211-212- | JJJF01000002.1 | 16 |
| ABC020041397 | 1 | 12 | -23-3-21- | JJKZ01000001.1 |  | 8 | -213-214-215-205- | JJKZ01000001.1 | 39 |
| ABC020041424 | 3 | 37 | cas+ CRISPR No | JJIJ01000001.1 |  | 55 | No cas | ND | 80 |
| ABC020043540 | 3 | 37 | cas+ CRISPR No | JJIA01000002.1 |  | 55 | No cas | ND | 80 |
| ABC020044010 | 3 | 37 | cas+ CRISPR No | JJFL01000001.1 |  | 55 | No cas | ND | 80 |
| ABC020044173 | 1 | 12 | -23-3-21- | JJGV01000001.1 |  | 8 | -213-214-215-205- | JJGV01000001.1 | 39 |
| ABC020044188 | 3 | 37 | cas+ CRISPR No | JJGU01000004.1 |  | 55 | No cas | ND | 80 |
| ABC020044193 | 3 | 37 | cas+ CRISPR No | JJGT01000001.1 |  | 55 | No cas | ND | 80 |
| ABC020044203 | 3 | 37 | cas+ CRISPR No | JJHW01000002.1 |  | 55 | No cas | ND | 80 |
| ABC020044412 | 3 | 37 | cas+ CRISPR No | JJFK01000002.1 |  | 55 | No cas | ND | 80 |
| ABC020046158 | 12 | 4 | -16-17- | JJJB01000001.1 |  | 22 | -250-251-212- | JJJB01000002.1 | 9 |
| ABC020046264 | 12 | 4 | -16-17- | JJGS01000001.1 |  | 42 | -208-224-225-209-210-211-212- | JJGS01000002.1 | 21 |
| ABC020046470 | 1 | 19 | -3-21- | JJGR01000003.1 |  | 8 | -213-214-215-205- | JJGR01000003.1 | 47 |
| ABC020046534 | 12 | 4 | -16-17- | JJIZ01000002.1 |  | 4 | -210-211-212- | JJIZ01000002.1 | 6 |
| ABC020046559 | 12 | 1 | -16- | JJJA01000002.1 |  | 42 | -208-224-225-209-210-211-212- | JJJA01000003.1 | 1 |
| ABC020046589 | 3 | 37 | cas+ CRISPR No | JJLA01000001.1 |  | 55 | No cas | ND | 80 |
| ABC020046688 | 1 | 14 | -23-77-21- | JJLB01000001.1 |  | 8 | -213-214-215-205- | JJLB01000001.1 | 41 |
| ABC020046986 | 1 | 14 | -23-77-21- | JJLC01000001.1 |  | 8 | -213-214-215-205- | JJLC01000001.1 | 41 |
| ABC020047076 | 3 | 37 | cas+ CRISPR No | JJHS01000002.1 |  | 55 | No cas | ND | 80 |
| ABC020047086 | 12 | 4 | -16-17- | JJJM01000001.1 |  | 29 | -250-251-252-208-224-225-209-210-211-212- | JJJM01000003.1 | 16 |
| ABC020047328 | 3 | 37 | cas+ CRISPR No | JJLD01000001.1 |  | 55 | No cas | ND | 80 |
| ABC020047395 | 3 | 37 | cas+ CRISPR No | JJFJ01000002.1 |  | 55 | No cas | ND | 80 |
| ABC020047506 | 12 | 3 | -16-16-17- | JJJL01000001.1 |  | 29 | -250-251-252-208-224-225-209-210-211-212- | JJJL01000001.1 | 4 |
| ABC020047619 | 3 | 37 | cas+ CRISPR No | JJHR01000001.1 |  | 55 | No cas | ND | 80 |
| ABC020047635 | 12 | 4 | -16-17- | JJJK01000001.1 |  | 42 | -208-224-225-209-210-211-212- | JJJK01000002.1 | 21 |
| ABC020047925 | 1 | 12 | -23-3-21- | JJFI01000002.1 |  | 8 | -213-214-215-205- | JJFI01000002.1 | 39 |
| ABC020047955 | 1 | 12 | -23-3-21- | JJJP01000001.1 |  | 8 | -213-214-215-205- | JJJP01000001.1 | 39 |
| ABC020047959 | 3 | 37 | cas+ CRISPR No | JJFH01000002.1 |  | 55 | No cas | ND | 80 |
| ABC020047977 | 1 | 12 | -23-3-21- | JJJX01000001.1 |  | 8 | -213-214-215-205- | JJJX01000001.1 | 39 |
| ABC020047993 | 12 | 4 | -16-17- | JJGQ01000004.1 |  | 28 | -250-251-252-208-224-224-224-224-224-225-209-210- | JJGQ01000005.1 | 15 |
| ABC020048184 | 12 | 4 | -16-17- | JJFG01000002.1 |  | 29 | -250-251-252-208-224-225-209-210-211-212- | JJFG01000003.1 | 16 |
| ABC020048262 | 12 | 4 | -16-17- | JJIY01000001.1 |  | 29 | -250-251-252-208-224-225-209-210-211-212- | JJIY01000002.1 | 16 |
| ABC020048387 | 1 | 12 | -23-3-21- | JJJY01000001.1 |  | 8 | -213-214-215-205- | JJJY01000001.1 | 39 |
| ABC020048395 | 3 | 37 | cas+ CRISPR No | JJGP01000002.1 |  | 55 | No cas | ND | 80 |
| ABC020048503 | 1 | 12 | -23-3-21- | JJJQ01000001.1 |  | 8 | -213-214-215-205- | JJJQ01000001.1 | 39 |
| ABC020048541 | 3 | 37 | cas+ CRISPR No | JJFF01000006.1 |  | 55 | No cas | ND | 80 |
| ABC020048543 | 12 | 4 | -16-17- | JJGO01000001.1 |  | 29 | -250-251-252-208-224-225-209-210-211-212- | JJGO01000001.1 | 16 |
| ABC020048606 | 12 | 4 | -16-17- | JJKN01000001.1 |  | 46 | -208-209-210-211-212- | JJKN01000001.1 | 25 |
| ABC020048885 | 3 | 37 | cas+ CRISPR No | JJII01000002.1 |  | 55 | No cas | ND | 80 |
| ABC020049250 | 1 | 12 | -23-3-21- | JJGN01000002.1 |  | 8 | -213-214-215-205- | JJGN01000002.1 | 39 |
| ABC020049540 | 3 | 37 | cas+ CRISPR No | JJIH01000004.1 |  | 55 | No cas | ND | 80 |
| ABC020049545 | 1 | 12 | -23-3-21- | JJJR01000001.1 |  | 8 | -213-214-215-205- | JJJR01000001.1 | 39 |
| ABC020050193 | 1 | 14 | -23-77-21- | JJLE01000001.1 |  | 8 | -213-214-215-205- | JJLE01000001.1 | 41 |
| ABC020050731 | 12 | 4 | -16-17- | JJGM01000001.1 |  | 25 | -250-251-252-225-209-210-211-212- | JJGM01000001.1 | 12 |
| ABC020050818 | 3 | 37 | cas+ CRISPR No | JJKO01000001.1 |  | 55 | No cas | ND | 80 |
| ABC020051161 | 1 | 12 | -23-3-21- | JJIL01000002.1 |  | 8 | -213-214-215-205- | JJIL01000002.1 | 39 |
| ABC020051269 | 3 | 37 | cas+ CRISPR No | JJLF01000001.1 |  | 55 | No cas | ND | 80 |
| ABC020052023 | 3 | 37 | cas+ CRISPR No | JJLG01000001.1 |  | 55 | No cas | ND | 80 |
| ABC020052203 | 12 | 4 | -16-17- | JJGL01000001.1 |  | 42 | -208-224-225-209-210-211-212- | JJGL01000002.1 | 21 |
| ABC020052211 | 12 | 4 | -16-17- | JJJO01000001.1 |  | 29 | -250-251-252-208-224-225-209-210-211-212- | JJJO01000001.1 | 16 |
| ABC020052216 | 12 | 2 | -17- | JJGK01000001.1 |  | 42 | -208-224-225-209-210-211-212- | JJGK01000002.1 | 3 |
| ABC020052218 | 1 | 12 | -23-3-21- | JJKP01000001.1 |  | 8 | -213-214-215-205- | JJKP01000001.1 | 39 |
| ABC020052291 | 1 | 12 | -23-3-21- | JJFE01000002.1 |  | 8 | -213-214-215-205- | JJFE01000003.1 | 39 |
| ABC020052309 | 1 | 12 | -23-3-21- | JJJS01000001.1 |  | 8 | -213-214-215-205- | JJJS01000001.1 | 39 |
| ABC020052313 | 1 | 12 | -23-3-21- | JJJT01000001.1 |  | 8 | -213-214-215-205- | JJJT01000001.1 | 39 |
| ABC020052375 | 1 | 12 | -23-3-21- | JJKQ01000001.1 |  | 8 | -213-214-215-205- | JJKQ01000001.1 | 39 |
| ABC020052378 | 3 | 37 | cas+ CRISPR No | JJHZ01000001.1 |  | 55 | No cas | ND | 80 |
| ABC020052420 | 3 | 37 | cas+ CRISPR No | JJGJ01000001.1 |  | 55 | No cas | ND | 80 |
| ABC020052519 | 3 | 37 | cas+ CRISPR No | JJIG01000001.1 |  | 55 | No cas | ND | 80 |
| ABC020052553 | 1 | 13 | -23-3-21-21- | JJKR01000001.1 |  | 8 | -213-214-215-205- | JJKR01000001.1 | 40 |
| ABC020052558 | 3 | 37 | cas+ CRISPR No | JJFC01000002.1 |  | 55 | No cas | ND | 80 |
| ABC020052877 | 1 | 12 | -23-3-21- | JJKS01000001.1 |  | 8 | -213-214-215-205- | JJKS01000001.1 | 39 |
| ABC020052898 | 1 | 12 | -23-3-21- | JJJZ01000001.1 |  | 8 | -213-214-215-205- | JJJZ01000001.1 | 39 |
| ABC020052980 | 1 | 12 | -23-3-21- | JJJU01000001.1 |  | 8 | -213-214-215-205- | JJJU01000001.1 | 39 |
| ABC020053060 | 12 | 4 | -16-17- | JJJE01000001.1 |  | 27 | -250-251-252-208-224-211-212- | JJJE01000002.1 | 14 |
| ABC020053240 | 3 | 37 | cas+ CRISPR No | JJIF01000001.1 |  | 55 | No cas | ND | 80 |
| ABC020053250 | 1 | 12 | -23-3-21- | JJKA01000001.1 |  | 8 | -213-214-215-205- | JJKA01000001.1 | 39 |
| ABC020054184 | 12 | 4 | -16-17- | JJJD01000001.1 |  | 42 | -208-224-225-209-210-211-212- | JJJD01000002.1 | 21 |
| ABC020054871 | 12 | 4 | -16-17- | JJGI01000001.1 |  | 24 | -250-251-225-209-210-211-212- | JJGI01000002.1 | 11 |
| ABC020054955 | 3 | 37 | cas+ CRISPR No | JJGH01000002.1 |  | 55 | No cas | ND | 80 |
| ABC020054973 | 12 | 4 | -16-17- | JJFB01000001.1 |  | 42 | -208-224-225-209-210-211-212- | JJFB01000002.1 | 21 |
| ABC020055897 | 3 | 37 | cas+ CRISPR No | JJIE01000001.1 |  | 55 | No cas | ND | 80 |
| ABC020056020 | 1 | 12 | -23-3-21- | JJIM01000002.1 |  | 8 | -213-214-215-205- | JJIM01000002.1 | 39 |
| ABC020056060 | 3 | 37 | cas+ CRISPR No | JJHV01000001.1 |  | 55 | No cas | ND | 80 |
| ABC020056064 | 3 | 37 | cas+ CRISPR No | JJID01000001.1 |  | 55 | No cas | ND | 80 |
| ABC020056068 | 1 | 12 | -23-3-21- | JJKB01000001.1 |  | 8 | -213-214-215-205- | JJKB01000001.1 | 39 |
| ABC020056181 | 1 | 12 | -23-3-21- | JJKT01000001.1 |  | 8 | -213-214-215-205- | JJKT01000001.1 | 39 |
| ABC020056765 | 1 | 12 | -23-3-21- | JJJV01000001.1 |  | 8 | -213-214-215-205- | JJJV01000001.1 | 39 |
| ABC020056794 | 12 | 4 | -16-17- | JJIX01000001.1 |  | 42 | -208-224-225-209-210-211-212- | JJIX01000002.1 | 21 |
| ABC020056883 | 1 | 12 | -23-3-21- | JJKU01000001.1 |  | 8 | -213-214-215-205- | JJKU01000001.1 | 39 |
| ABC020056884 | 12 | 4 | -16-17- | JJJJ01000002.1 |  | 42 | -208-224-225-209-210-211-212- | JJJJ01000002.1 | 21 |
| ABC020056885 | 1 | 12 | -23-3-21- | JJGG01000003.1 |  | 8 | -213-214-215-205- | JJGG01000003.1 | 39 |
| ABC020056891 | 3 | 37 | cas+ CRISPR No | JJHU01000002.1 |  | 55 | No cas | ND | 80 |
| ABC020056894 | 12 | 4 | -16-17- | JJJI01000001.1 |  | 29 | -250-251-252-208-224-225-209-210-211-212- | JJJI01000001.1 | 16 |
| ABC020056898 | 3 | 37 | cas+ CRISPR No | JJHT01000003.1 |  | 55 | No cas | ND | 80 |
| ABC020057168 | 3 | 37 | cas+ CRISPR No | JJHY01000001.1 |  | 55 | No cas | ND | 80 |
| ABC020057192 | 3 | 37 | cas+ CRISPR No | JJHP01000003.1 |  | 55 | No cas | ND | 80 |
| ABC020057288 | 1 | 12 | -23-3-21- | JJIK01000001.1 |  | 8 | -213-214-215-205- | JJIK01000001.1 | 39 |
| ABC020057442 | 1 | 9 | -23-23-21- | JJJW01000001.1 |  | 8 | -213-214-215-205- | JJJW01000001.1 | 36 |
| ABC020057449 | 3 | 37 | cas+ CRISPR No | JJHX01000002.1 |  | 55 | No cas | ND | 80 |
| ABC020058759 | 12 | 4 | -16-17- | JJGF01000002.1 |  | 42 | -208-224-225-209-210-211-212- | JJGF01000002.1 | 21 |
| ABC020059502 | 1 | 12 | -23-3-21- | JJKC01000001.1 |  | 8 | -213-214-215-205- | JJKC01000001.1 | 39 |
| ABC020060220 | 12 | 4 | -16-17- | JJIW01000002.1 |  | 42 | -208-224-225-209-210-211-212- | JJIW01000002.1 | 21 |
| ABC020060777 | 12 | 4 | -16-17- | JJJH01000001.1 |  | 29 | -250-251-252-208-224-225-209-210-211-212- | JJJH01000001.1 | 16 |
| ABC020060793 | 3 | 37 | cas+ CRISPR No | JJFA01000003.1 |  | 55 | No cas | ND | 80 |
| ABC020061424 | 1 | 12 | -23-3-21- | JJKD01000001.1 |  | 8 | -213-214-215-205- | JJKD01000001.1 | 39 |
| ABC020062474 | 12 | 4 | -16-17- | JJIV01000001.1 |  | 43 | -208-224-225-209-210-212- | JJIV01000002.1 | 22 |
| ABC020062601 | 12 | 4 | -16-17- | JJIU01000002.1 |  | 30 | -250-251-252-208-224-225-209-224-225-209-210-211-212- | JJIU01000002.1 | 17 |
| ABC020064181 | 12 | 4 | -16-17- | JJJC01000002.1 |  | 42 | -208-224-225-209-210-211-212- | JJJC01000002.1 | 21 |
| BJCYGAS15 | 12 | 4 | -16-17- | ALKD01000019.1 |  | 26 | -250-251-252-208-211-212- | ALKD01000007.1 | 13 |
| DSM 20565 | 1 | 37 | cas+ CRISPR No | ATXR01000001.1 |  | 8 | -213-214-215-205- | ATXR01000006.1 | 73 |
| GA40377 | 28 | 23 | -36-37-17-28- | AWTK01000017.1 |  | 17 | -229-230- | AWTK01000033.1 | 52 |
| GA41345 | 1 | 12 | -23-3-21- | AUOY01000075.1 |  | 8 | -213-214-215-205- | AUOY01000110.1 | 39 |
| HLJGAS12011 | 12 | 4 | -16-17- | ALKE01000011.1 |  | 47 | -208-209-211-212- | ALKE01000012.1 | 26 |
| SP1-LAU | 12 | 4 | -16-17- | AYPA01000012.1 |  | 48 | -208-209-212- | AYPA01000006.1 | 27 |
| SP4-LAU | 28 | 23 | -36-37-17-28- | AWPB01000002.1 |  | 17 | -229-230- | AWPB01000007.1 | 52 |
| SP5-LAU | 1 | 14 | -23-77-21- | AWPC01000001.1 |  | 8 | -213-214-215-205- | AWPC01000004.1 | 41 |
| UTMEM-1 | 28 | 23 | -36-37-17-28- | AVCF01000047.1 |  | 17 | -229-230- | AVCF01000054.1 | 52 |
| UTSW-2 | 28 | 23 | -36-37-17-28- | AVCG01000066.1 |  | 17 | -229-230- | AVCG01000073.1 | 52 |

* Each Number indicates a specific spacer. The “-” indicates the repeat sequence. “No cas” indicates that there is no *cas* genes cassette. “cas+ CRISPR NO” indicates the strain had a *cas* cassette, but no CRISPR array. The lead sequences are located at the left side of each spacer content.

^#^ “ND” indicates that *cas* cassettes cannot be identified by BLASTN according to description in materials and methods.
