# Supplementary material for: Clustered Regularly Interspaced Short Palindromic Repeats Are emm Type-Specific in Highly Prevalent Group A Streptococci
Source: PLoS One. 2015 Dec 28;10(12):e0145223. doi: 10.1371/journal.pone.0145223 (PMC4692479; doi:10.1371/journal.pone.0145223)
Supplement: S3 Table — (DOCX) [file pone.0145223.s004.docx]

**S3 Table.** The *emm* type, accession number, and CRISPR information of strains with complete genomes used in this study

|  |  |  |  | CRISPR01 |  |  | CRISPR02 |  |
| --- | --- | --- | --- | --- | --- | --- | --- | --- |
| Strain | Accession  number | *emm* type | Type | Spacer content * |  | Type | Spacer content * | CRISPRa type |
| A20 | CP003901.1 | 1 | 12 | -23-3-21- |  | 8 | -213-214-215-205- | 39 |
| SF370 | AE004092.1 | 1 | 25 | -44-19-3-20-21-22- |  | 7 | -213-214-215- | 54 |
| MGAS5005 | CP000017.1 | 1 | 12 | -23-3-21- |  | 8 | -213-214-215-205- | 39 |
| 476 | AP012491.1 | 1 | 12 | -23-3-21- |  | 8 | -213-214-215-205- | 39 |
| MGAS315 | AE014074.1 | 3 | 37 | cas+ CRISPR No |  | 55 | No cas | 80 |
| SSI-1 | BA000034.2 | 3 | 37 | cas+ CRISPR No |  | 55 | No cas | 80 |
| STAB902 | CP007041.1 | 3 | 37 | cas+ CRISPR No |  | 55 | No cas | 80 |
| MGAS10750 | CP000262.1 | 4 | 37 | cas+ CRISPR No |  | 13 | -226-227-228-229-230- | 76 |
| MGAS2096 | CP000261.1 | 12 | 4 | -16-17- |  | 44 | -208-224-209-210-211-212- | 23 |
| MGAS9429 | CP000259.1 | 12 | 4 | -16-17- |  | 42 | -208-224-225-209-210-211-212- | 21 |
| HKU16 | AFRY01000001.1 | 12 | 4 | -16-17- |  | 26 | -250-251-252-208-211-212- | 13 |
| MGAS6180 | CP000056.1 | 28 | 23 | -36-37-17-28- |  | 2 | -229- | 51 |

* Each Arabic number indicates a specific spacer. The “-” indicates the repeat sequence. “No cas” indicates that there is no *cas* gene cassette. “cas+ CRISPR NO” indicates the strain had a *cas* cassette, but no CRISPR array. The lead sequences are located at the left side of each spacer content.
