# Supplementary material for: Clustered Regularly Interspaced Short Palindromic Repeats Are emm Type-Specific in Highly Prevalent Group A Streptococci
Source: PLoS One. 2015 Dec 28;10(12):e0145223. doi: 10.1371/journal.pone.0145223 (PMC4692479; doi:10.1371/journal.pone.0145223)
Supplement: S4 Table — (DOCX) [file pone.0145223.s005.docx]

**S4 Table.** Spacer sequences from CRISPR01 and CRISPR02 and their relationship to *emm* type

| Name | sequence | Length | *emm* types containing this spacer | |
| --- | --- | --- | --- | --- |
| **CRISPR01** | | | |  |
| 3 | AGGAATATCCGCGATAATTAATTGCGCTCT | 30 | 1 | |
| 16 | GATTGACCACAACATCCAACGCTTAGGTTAT | 31 | 12 | |
| 17 | AATAGAGCTAGACAAAAAATTGAGTTTGAC | 30 | 12,28 | |
| 18 | CTTCGTCAAAAGTCAAAGGGAAAAGGAAGA | 30 | 1 | |
| 19 | TTATATGAACATAACTCAATTTGTAAAAAA | 30 | 1 | |
| 20 | AGTGCCGAGGAAAAATTAGGTGCGCTTGGC | 30 | 1 | |
| 21 | TAAATTTGTTTAGCAGGTAAACCGTGCTTT | 30 | 1 | |
| 22 | TTCAGCACACTGAGACTTGTTGAGTTCCAT | 30 | 1 | |
| 23 | GGGTGGTTGGCTGACGCATCGCAATATTAA | 30 | 1 | |
| 28 | GACATATTAACGTCCTTTCTCCTGCTTTCC | 30 | 28 | |
| 36 | TGCTCCAGATGGATTTTTAAACTGATTATT | 30 | 28 | |
| 37 | TAGAAATTGACAATGCTTTTTCTTTTGTCT | 30 | 28 | |
| 44 | TGCGCTGGTTGATTTCTTCTTGCGCTTTTT | 30 | 1 | |
| 77 | AGGAATATCCGCAATAATTAATTGCGCTCTGTT | 33 | 1 | |
| **CRISPR02** | | | |  |
| 205 | TGTCCGCATACCTTGATTTGAGCGAGTAAACTC | 33 | 1 | |
| 208 | ATCGATTTTGCAGATAAAAGGAAACATAGAGTTC | 34 | 12 | |
| 209 | AGATTCGGCTAGATATTCTAAAAAATCGATAAAGC | 35 | 12 | |
| 210 | TCCAACTTTTGTAAAAGTAGAATTTGCTACGTTTG | 35 | 12 | |
| 211 | ATTATTTTATTAAAGTTATCACGTAAATTTTGCAA | 35 | 12 | |
| 212 | CATTATATGAACAATGCCTTTGCGGAATTAGTT | 33 | 12 | |
| 213 | AAAAAGCATATCACGAAAATCACCAATTACATCAG | 35 | 1 | |
| 214 | AAGTCATTTTCTGCTTCTGCTAGGTTTGCTTTA | 33 | 1 | |
| 215 | TTTTTACTTTGATTACATCCCGCAATGTCACAGC | 34 | 1 | |
| 224 | AATTTCATCTCCTGCATCTTGATCAGTTAGGGTTAC | 36 | 12 | |
| 225 | ATGAACTAGGACATATTGAACATGATTCTGGCCAAT | 36 | 12 | |
| 226 | TACTAGCGTTGAGATAGCCGGTATTAATCTTACC | 34 | 4 | |
| 227 | ATTTCGTCACCTCCTCAATCAATAATAGAGTCA | 33 | 4 | |
| 228 | CTAAGATGATACCAGTTACAATACCGATTTTAAGC | 35 | 4 | |
| 229 | AAATAATTACATGACTAGCCAATACACCCACATA | 34 | 4,28 | |
| 230 | TTCATGAGCTTCTTTACTCTCAAAGTAAGAGTG | 33 | 4,28 | |
| 237 | CCAAAGGCTGAACAAGGTGTTCGTAATCTCGTG | 33 | 12 | |
| 250 | TCACATAGCAAAATGCGGTTATTGTGGTGCCCCTT | 35 | 12 | |
| 251 | GCGCATAGCTTGACTAGGCTTGTATTTATTACCG | 34 | 12 | |
| 252 | TATAATGATGGCACACTCTTCACCAATTGATACA | 34 | 12 | |
| 289 | ATGAACTAGGACATATTGAACATGATTTAGGGTAAT | 36 | 12 | |
