# Supplementary material for: Clustered Regularly Interspaced Short Palindromic Repeats Are emm Type-Specific in Highly Prevalent Group A Streptococci
Source: PLoS One. 2015 Dec 28;10(12):e0145223. doi: 10.1371/journal.pone.0145223 (PMC4692479; doi:10.1371/journal.pone.0145223)
Supplement: S5 Table — (DOCX) [file pone.0145223.s006.docx]

**S5 Table.** Adjusted Wallace coefficients and jackknife pseudo-value 95% confident interval (CI) for the *emm*, CRISPRa, CRISPR01, and CRISPR02 types among all foreign and local strains

|  | Adjusted Wallace coefficient (95% CI) | | | |
| --- | --- | --- | --- | --- |
| Typing method | *emm* | CRISPR01 | CRISPR02 | CRISPRa |
| *emm* |  | 0.749  (0.665-0.831) | 0.386  (0.289-0.480) | 0.266  (0.198-0.331) |
| CRISPR01 | 0.743  (0.669-0.818) |  | 0.233  (0.176-0.288) | 0.264  (0.205-0.321) |
| CRISPR02 | 1.000  (1.000-1.000) | 0.606  (0.485-0.723) |  | 0.688  (0.586-0.786) |
| CRISPRa | 1.000  (1.000-1.000) | 1.000  (1.000-1.000) | 1.000  (1.000-1.000) |  |
